# Silent Threat: Multi‐Organ Failure in Neonatal Scrub Typhus Without Traditional Markers

**DOI:** 10.1002/ccr3.71352

**Published:** 2025-11-07

**Authors:** Li Hu, Shuyan Li, Fangjian Gao, Shiguang Diao, Xiaoyan Liu, Jianwu Qiu

**Affiliations:** ^1^ Department of Neonatology, Yuebei People's Hospital Guangdong Medical University Shaoguan China; ^2^ Guangdong Province Critical Newborn Rescue Center (North Guangdong) Shaoguan China

**Keywords:** multiple organ failure, neonates, rickettsial disease, scrub typhus, tsutsugamushi

## Abstract

Scrub typhus, an acute zoonotic disease from *Orientia tsutsugamushi* , is uncommon in newborns and presents atypical symptoms. Untimely diagnosis and treatment can lead to a prolonged and potentially fatal course. Early diagnosis and treatment are essential for better patient outcomes. Metagenomic next‐generation sequencing can rapidly and accurately diagnose pathogens, aiding precise treatment.

## 1 Introduction

Tsutsugamushi disease, also known as scrub typhus zoonotic disease in humans, is a naturally occurring disease caused by *Orientia tsutsugamushi*. Rodents serve as crucial reservoir hosts for trombiculid mites, whereas the larvae of these mites act as the primary vectors for transmission [1]. The incidence of this disease is low among children, and even lower in neonates. Among all patients with scrub typhus, preschool children account for 6.38% [2], neonates account for only 1.3% [3], and neonatal scrub typhus presenting with multi‐organ failure is even rarer. Moreover, its clinical manifestations in newborns are atypical, which easily leads to missed and misdiagnosed cases that are life‐threatening [4].

This report details a rare sporadic case of scrub typhus in a neonate from Northern Guangdong within 2 weeks post‐birth. In this case, the infant presented without scabs or ulcers, had negative Weil‐Felix test results, and experienced multiple organ failure. The disease's etiology could not be determined by conventional tests, and the diagnosis of *O. tsutsugamushi* infection was ultimately confirmed through metagenomic next‐generation sequencing (mNGS). This case was characterized by its rare onset in the neonatal period, atypical clinical manifestations, a complex diagnostic process, severe multi‐organ failure, and the application of mNGS as a new technology that is particularly noteworthy. It holds significant clinical reference value for enhancing medical staff's ability to recognize the early stage of scrub typhus with atypical clinical manifestations, enabling timely and effective treatment, and improving the prognosis and regression of the disease. Moreover, this case study offers new insights and directions for future research and clinical practice in related fields.

## 2 Case Presentation/Examination

A 14‐day‐old male infant was admitted to our hospital on October 24 due to fever and tachypnea. The infant was the fourth fetus and second delivery for the mother and was born by cesarean section at 35 weeks and 4 days gestation, with a birth weight of 2.9 kg. The infant was discharged from the hospital with his mother 5 days after birth. On the 14th day after birth, the infant developed a fever up to 38.5°C, accompanied by tachypnea. The infant had received antipyretic treatment at a district hospital and was then referred to our hospital. The mother denied any infections during pregnancy, and the family denied any recent infections. Upon admission, physical examination revealed: temperature 36.5°C, pulse 130 beats per minute, respiration 52 breaths per minute, and blood pressure 68/35 mmHg. The infant was conscious, with moderate jaundice of the skin and sclera. The skin was intact without eschars or ulcers. No enlarged superficial lymph nodes were palpable. The anterior fontanelle was flat and soft, and the pupillary light reflex was brisk. The infant had tachypnea with slight tracheal tug. The lungs had coarse breath sounds and a few coarse crackles. The heart rate was 130 beats per minute, with a regular rhythm and no murmurs. The abdomen was slightly distended, with normal bowel sounds. The muscle strength and tone of the limbs were normal, and the neonatal reflexes were present.

## 3 Methods

Upon admission, the infant's transcutaneous oxygen saturation was 88%–93%, and he received high‐flow nasal cannula oxygen therapy and anti‐infection treatment with piperacillin‐tazobactam. Blood culture tests were conducted prior to the administration of antibiotics. Additionally, a series of comprehensive tests were performed, including assessments of inflammatory markers, liver function, renal function, and coagulation parameters. The specific results are detailed in Table 1. The chest X‐ray indicated bilateral pneumonia. Viral DNA testing, viral antibody testing, as well as routine, biochemical, and smear examinations of cerebrospinal fluid (CSF) revealed no abnormalities.

**TABLE 1:** Indexes of inflammatory indices, liver function, renal function, coagulation function.

Content items	1d	2d	4d	5d	7d	8d	14d	15d
WBC (*10^9^/L)	11.17	7.58	8.06	14.83	43.99	33.20	40.98	35.37
PLT (*10^9^/L)	89	82	24	28	53	75	103	33
CRP (mg/L)	19.49	77.24	141.77	106.25	79.11	61.07		127.67
PCT (ng/ml)	1.370	1.564	1.331	1.080				
ALT (U/L)	76			51.30	37.7	1051	54.8	52.6
AST (U/L)	262.20			124.60	228.9	8160.9	153,9	102.5
BUN (mmol/L)	2.9			1.91	3.52	7.39	5.33	7.1
Cr (Umol/L)	38.9			14.7	22.7	66.3	33.7	39
PT (s)				18.1	> 100	32.8	16.9	18.3
FIB (g/L)				0.69	< 0.5	< 0.5	1.14	1.02

Abbreviations: ALT, alanine aminotransferase; AST, aspartate aminotransferase; BUN, blood urea nitrogen; Cr, creatinine; CRP, C‐reactive protein; FIB, fibrinogen; PCT, procalcitonin; PLT, platelets; PT, prothrombin time; WBC, white blood cell count.

On the second day post‐admission, the infant's condition worsened, exhibiting a poor response, recurrent fever, a marbled skin pattern, abdominal distension, and an elevated CRP level compared with the initial admission. Consequently, blood tandem mass spectrometry and urinary organic acid tests were further perfected. The treatment was then switched to cefoperazone‐sulbactam and vancomycin for anti‐infection purposes.

**FIGURE 1:**
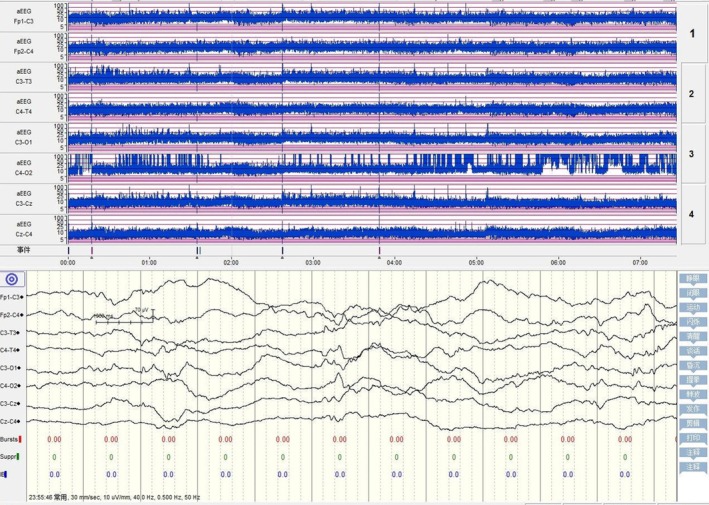
Amplitude‐integrated EEG on the fourth day of admission: Immature sleep–wake cycle (SWC) and mildly abnormal neonatal EEG (mildly discontinuous background activity).

By the fourth day, the infant's condition had further deteriorated, with recurrent fever, a diminished mental response, worsening tachypnea, fluctuating oxygen saturation, frothing at the mouth, increased abdominal distension, a significant drop in platelet count, and a marked increase in CRP. Abdominal ultrasound showed no abnormalities in the liver biliary system, urinary system, and abdominal aorta. Echocardiography detected patent ductus arteriosus, patent foramen ovale, and mild tricuspid valve insufficiency. Cranial ultrasound indicated widening of the bilateral ventricles. Electroencephalography showed mild neonatal abnormalities (Figure 1). A second blood culture was immediately performed; the treatment regimen was altered to include meropenem and vancomycin for combined anti‐infection therapy, along with a platelet transfusion.

On the fifth day, the infant's condition continued to decline, with respiratory failure, hard skin edema, hypotension, severe mixed acidosis, hyperkalemia, hyponatremia, oliguria, and damage to liver, kidney, and heart function. Blood tandem mass spectrometry showed elevated levels of multiple amino acids, and urine gas chromatography revealed ketonuria and increased 4‐hydroxyphenyl‐L‐lactate. The infant was intubated and placed on a ventilator for assisted ventilation, administered vasopressors to elevate blood pressure, hydrocortisone for anti‐inflammatory purposes, intravenous immunoglobulin for enhanced support, plasma transfusion to improve coagulation function, and treatment to maintain electrolyte stability and protect organ function. Further investigation into the medical history revealed that the infant resided in a rural area, with family members having a history of contact with tea gardens and other vegetation. Blood and cerebrospinal fluid metagenomic next‐generation sequencing were promptly conducted, and a Weil‐Felix test was performed. Given that the blood tandem mass spectrometry results did not rule out the possibility of genetic diseases such as mitochondrial disorders, whole‐exome sequencing was decided upon.

On the seventh day of admission, the first blood culture result was reported as negative, and the Weil‐Felix test was also negative. The results of the metagenomic next‐generation sequencing revealed a high sequence of *O. tsutsugamushi* in both blood and cerebrospinal fluid samples. After confirming the diagnosis of neonatal scrub typhus, a multidisciplinary MDT consultation was held with the infectious disease department, and the family was informed. With the family's informed consent, azithromycin (10 mg/kg) was administered for anti‐infection treatment, and CRRT was initiated.

On the ninth day of admission, despite undergoing 2 days of treatment with azithromycin, the infant's condition did not significantly improve. The Weil‐Felix test was rechecked. The family was informed once more, and following consultation with the pharmacy department and obtaining the family's consent, chloramphenicol (25 mg/kg, divided into two intravenous infusions) was administered for anti‐infection treatment.

On the tenth day of admission, the second Weil‐Felix test result was negative, and the second blood culture report was also negative. Repeated cranial ultrasound revealed widened bilateral ventricles and increased echogenicity of the brain parenchyma. Ultrasound examination of the urinary system showed increased echogenicity of the renal parenchyma and the presence of ascites.

On the fourteenth day of admission, a re‐examination of inflammatory markers, liver function, kidney function, and coagulation function indicated some improvement; however, the infant remained unconscious.

**FIGURE 2:**
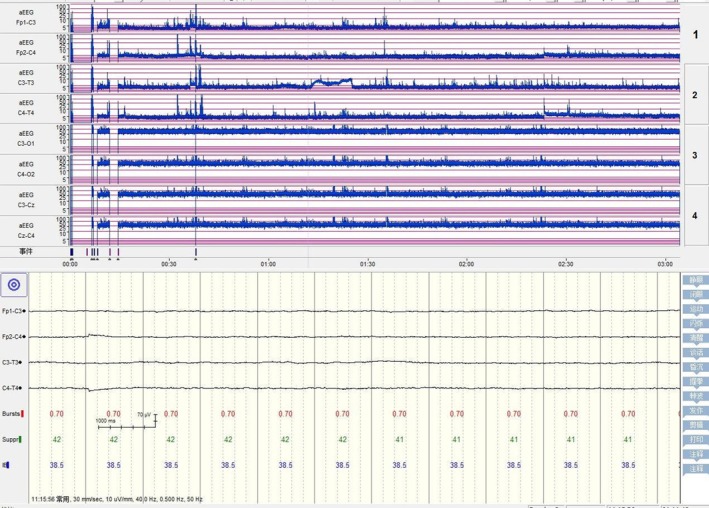
Amplitude‐integrated EEG on the 15th day of admission: Electrical silence, flat waves, absence of SWC, and severely abnormal amplitude‐integrated EEG changes.

On the fifteenth day post‐admission, the repeated electroencephalogram revealed electrical silence with flat waves (Figure 2). The family decided to discontinue treatment.

## 4 Outcome and Follow‐Up

Upon confirming the diagnosis, the infant was aggressively treated with anti‐infection therapy, supportive care, and continuous renal replacement therapy (CRRT), which led to improved blood indicators. Despite these efforts, the infant remained in a persistent coma, prompting the family to decide to discontinue treatment. Follow‐up after discharge indicated that the infant had passed away on the day of discharge. Half a month after discharge, the results of the whole‐exome sequencing were reported, indicating that no pathogenic variants were detected.

## 5 Discussion

Scrub typhus is a zoonotic disease caused by *O. tsutsugamushi* , also known as rickettsiae [5]. It is an acute natural zoonosis transmitted through the bite of trombiculid mites. The infection begins when the rickettsiae proliferate at the site of the bite, causing skin damage. Subsequently, they enter the bloodstream, leading to tsutsugamushi rickettsiae bacteremia. The rickettsiae then grow and reproduce within vascular endothelial cells and the mononuclear‐macrophage system, producing toxins that result in inflammation of small and surrounding blood vessels throughout the body [6].

The incubation period of scrub typhus is approximately 4–21 days. It predominantly occurs in farmers, whose typical clinical symptoms include fever, eschars or ulcers, lymphadenopathy, and skin rash [7]. However, neonatal infections are rare, and the clinical manifestations are atypical, more complex, with rapid disease progression and multiple complications. In neonates with scrub typhus, 75% of the cases are infected after birth, whereas 25% are transmitted vertically from mother to newborn. All affected neonates present with fever, hepatosplenomegaly, and thrombocytopenia (100%). Only 8% of the cases have eschars. The most common complications include shock (66.7%), respiratory failure (41.7%), disseminated intravascular coagulation (DIC, 33.3%), and multiple organ dysfunction syndrome (MODS, 25%), with a mortality rate of 25% [3]. Guangdong Province is a high‐incidence area for scrub typhus, with nearly one‐third of the cases in China occurring there. The first case of human infection with *O. tsutsugamushi* in China was identified in Guangdong Province in 1948 [8], where scrub typhus occurred throughout the entire year but was most prevalent from May to November [9].

The infant in this case presented with fever and tachypnea as the initial symptoms. No eschars or ulcers were detected on the skin and mucous membranes. There was no palpable enlargement of superficial lymph nodes, liver, or spleen. The clinical manifestations were nonspecific, but the condition progressed rapidly, resulting in multi‐organ failure. The onset of the disease occurred in early November, coinciding with the local epidemic season for scrub typhus. Upon further inquiry into the medical history, it was revealed that the infant's family had a history of contact with vegetation in tea gardens. Studies have confirmed that the northern Guangdong mountainous area is a natural hotspot for scrub typhus [10]. In the wilderness and jungles of northern Guangdong, there are many places suitable for the growth of trombiculid larvae. Contact with tea garden vegetation by family members could potentially introduce trombiculid mites into the home, and neonates could be inadvertently infected.

If a patient exhibits typical clinical manifestations, diagnosis is typically straightforward and the mortality rate is low. However, for those without such manifestations, failure to treat promptly may result in prolonged illness and potentially life‐threatening conditions. The mortality rate for severe cases of scrub typhus, or those resulting from improper treatment or misdiagnosis, can reach 30%–70% [11]. The World Health Organization has pointed out that scrub typhus is one of the most easily missed infectious diseases at present [12]. Therefore, accurate and swift laboratory diagnosis is crucial for confirming and treating scrub typhus. The absence of a specific diagnostic method and knowledge about scrub typhus is a common issue in China. At present, the diagnosis of scrub typhus mainly depends on serological tests, among which the most commonly used serological test method is the Weil‐Felix test. The Weil‐Felix test is an agglutination test using Proteus OXK, which has common antigens with *O. tsutsugamushi* , as the antigen. The Weil‐Felix test is simple and easy to operate, but its sensitivity and specificity are relatively low [13]. For suspected cases of scrub typhus with negative traditional serology, alternative serological methods such as indirect immunofluorescence assay (IFA) or enzyme‐linked immunosorbent assay (ELISA) should be considered to enhance the diagnostic rate. Additionally, direct detection methods like PCR are more timely, and qPCR can be utilized for diagnosing early disease, specifically those of less than 7 days duration [14]. Metagenomic next‐generation sequencing (mNGS) is a novel pathogen—detection technique that can directly perform high‐throughput sequencing on the nucleic acids (DNA or RNA) of infected specimens [13]. It is capable of rapidly and objectively detecting a wide range of pathogenic microorganisms in clinical specimens, including viruses, bacteria, fungi, parasites, spirochetes, and rickettsiae. It does not rely on the traditional culture of pathogenic microorganisms and is especially suitable for the diagnosis of critical, severe, and difficult‐to‐diagnose infections [15, 16]. Currently, the use of mNGS for the detection of scrub typhus is not yet widespread. However, small‐sample studies have shown that the sensitivity of mNGS detection for scrub typhus is 100%. In the early stage of the disease, for patients without typical eschars or ulcers, the diagnostic value of mNGS is much higher than that of the Weil‐Felix reaction and various serological antibody methods. In addition, mNGS can also be utilized for the rapid differentiation between *O. tsutsugamushi* and other pathogen infections [17, 18]. Clinical centers should select appropriate detection methods based on the specific conditions of their own laboratories and the actual conditions of patients.

The infant in this case presented with a severe condition that progressed rapidly following admission. The Weil‐Felix test was conducted twice during the first and second weeks of the disease course, both resulting in a negative outcome and failing to identify the pathogen. On the fifth day of the disease course, mNGS was employed to test blood and cerebrospinal fluid samples. The results, which were returned after 48 h, indicated that the number of *O. tsutsugamushi* sequences in the blood sample reached 12,779, and in the cerebrospinal fluid, it reached 8839. Consequently, the infant was diagnosed with neonatal scrub typhus. Furthermore, despite the administration of multiple antibiotics, high‐sequence *O. tsutsugamushi* nucleic acids were still detected, and other febrile diseases with similar symptoms were simultaneously ruled out. If advanced detection methods such as qPCR are used, the time for a definitive diagnosis in infants could be advanced by more than 1 day. However, it is regrettable that our laboratory has not yet launched PCR detection for scrub typhus.

*Orientia tsutsugamushi* is a strictly intracellular microorganism that stains negative with Gram stain. Antibiotics that cannot enter the cells are ineffective against scrub typhus [19]. Consequently, the infant in this case was treated with β‐lactam antibiotics such as piperacillin‐tazobactam, cefoperazone‐sulbactam, and carbapenem antibiotic meropenem, yet the condition failed to improve. Due to the rapid progression of the disease and the occurrence of multi‐organ failure, upon confirming the diagnosis of scrub typhus, the infant was treated with sensitive antibiotics such as azithromycin and chloramphenicol, as well as comprehensive treatments. Although the infant's blood indicators, including liver function, kidney function, coagulation function, and acidosis, showed improvement, the infant remained in a coma. Eventually, the family requested and signed to give up treatment. The infant died shortly after discharge. Doxycycline, a derivative of tetracycline, was once considered contraindicated for pregnant women and children under the age of 8 due to potential risks such as tooth discoloration, inhibited bone growth, and teratogenic effects. Nevertheless, a systematic literature review has indicated that for the treatment of scrub typhus, doxycycline should be the first‐line treatment for both pregnant women and children [20]. Other studies have found that early use of doxycycline can improve prognosis. If scrub typhus is highly suspected and the condition is severe (such as multiple organ dysfunction syndrome), doxycycline should be prioritized, even in neonates [21]. Some literature has reported that combination therapy with intravenous doxycycline and azithromycin is a better therapeutic option for the treatment of severe scrub typhus than monotherapy with either drug [22]. The first antibiotic employed to treat scrub typhus was chloramphenicol; however, it proved to be less effective in severe cases and is now seldom used [23]. In this instance, following the ineffectiveness of azithromycin treatment, chloramphenicol was administered for anti‐infection purposes, under the supervision of the pharmacy department and with the family's consent. Post‐treatment, blood indicators, including liver and kidney function as well as coagulation function, showed improvement; nevertheless, the infant remained in a coma. In the future, for similar severe neonatal cases, doxycycline (or in combination with azithromycin) should be prioritized and administered as early as possible after a thorough risk–benefit assessment and obtaining informed consent.

In the existing literature, scrub typhus cases are predominantly observed in adults, particularly among middle‐aged individuals. Neonatal scrub typhus is exceedingly rare, particularly when it presents within 2 weeks post‐birth, thereby significantly complicating diagnosis and treatment. The classical clinical manifestations of scrub typhus encompass fever, eschar or ulcer, rash, as well as hepatosplenomegaly and lymphadenopathy. The infant in this case did not exhibit eschar or ulcer, and the outcomes of two Weil‐Felix tests were negative, characterizing it as an atypical case susceptible to misdiagnosis and missed diagnosis. Instances of scrub typhus complicated by multi‐organ failure carry a high mortality rate, especially for patients who do not receive timely treatment or whose condition deteriorates swiftly. The infant in this case experienced multi‐organ failure with rapid disease progression and a poor prognosis. Currently, the use of mNGS in diagnosing scrub typhus is not widely adopted, yet this case highlights its potential benefits in managing challenging cases.

In the diagnosis and treatment process, detailed and high‐quality medical history taking, as well as comprehensive and systematic physical examination, is particularly important. However, for cases with atypical clinical manifestations, the risk of misdiagnosis and missed diagnosis is high. As the disease progresses, patients may suffer from multi‐organ failure and even face life‐threatening conditions. Therefore, early diagnosis and treatment are crucial for improving the prognosis and outcomes of patients. When facing clinical infectious diseases, especially those difficult‐to‐diagnose severe infections that are hard to confirm in the early stage, the early use of mNGS technology can provide rapid and accurate pathogen diagnosis, thus offering strong support for precise treatment.

## 6 Conclusion

In this report, we present a case of sporadic neonatal scrub typhus infection without eschars or ulcers, a negative Weil‐Felix test, complicated by multiple organ failure. The infant underwent routine examination, and no etiology was identified. The history was further investigated; the infant resided in a rural area, and the family members had a history of exposure to grasses and trees, including tea gardens. Finally, the *O. tsutsugamushi* infection was confirmed by mNGS. After the diagnosis was clarified, the infant was treated with aggressive anti‐infection, support, and CRRT. All blood indicators improved, but the infant continued to be in a coma, and the family opted to cease treatment.

For difficult‐to‐diagnose severe infectious diseases that cannot be confirmed early, the early use of mNGS can provide rapid and accurate pathogen diagnosis, supporting precise treatment.

## Author Contributions

**Li Hu:** conceptualization, data curation, investigation, writing – original draft. **Shuyan Li:** data curation, investigation, writing – original draft. **Fangjian Gao:** data curation, investigation, visualization. **Shiguang Diao:** resources, validation. **Xiaoyan Liu:** resources, validation. **Jianwu Qiu:** conceptualization, funding acquisition, project administration, supervision, writing – review and editing.

## Ethics Statement

This study has been approved by the Ethics Committee of Yuebei People's Hospital (approval number: YBEC‐KY‐2023‐127).

## Consent

In accordance with the journal's patient consent policy, written informed consent was obtained from the infant's parents for the publication of this report.

## Conflicts of Interest

The authors declare no conflicts of interest.

## Data Availability

The data supporting the findings of this case report are available from the corresponding author upon reasonable request.
